# Combining Colistin with Furanone C-30 Rescues Colistin Resistance of Gram-Negative Bacteria *in Vitro* and *in Vivo*

**DOI:** 10.1128/Spectrum.01231-21

**Published:** 2021-11-03

**Authors:** Ying Zhang, Yishuai Lin, Xiaodong Zhang, Liqiong Chen, Chunyan Xu, Shixing Liu, Jianming Cao, Xiangkuo Zheng, Huaiyu Jia, Lijiang Chen, Tieli Zhou

**Affiliations:** a Department of Clinical Laboratory, The First Affiliated Hospital of Wenzhou Medical University, Wenzhou, China; b Department of Medical Lab Science, School of Laboratory Medicine and Life Science, Wenzhou Medical University, Wenzhou, China; University of Guelph

**Keywords:** Gram-negative bacteria, colistin-resistant, furanone C-30, biofilm, synergy effect, multidrug resistance

## Abstract

The spread of multidrug-resistant (MDR) Gram-negative bacteria (GNB) has led to serious public health problems worldwide. Colistin, as a “last resort” for the treatment of MDR bacterial infections, has been used significantly in recent years and has led to the continuous emergence of colistin-resistant strains. In this study, we aimed to investigate the synergistic effect on the antimicrobial and antibiofilm activities of a colistin/furanone C-30 combination against colistin-resistant GNB *in vitro* and *in vivo*. According to antimicrobial resistance profiles, most of the colistin-resistant strains we collected showed MDR phenotypes. The checkerboard method and time-kill curve showed that the combination with furanone C-30 increases the antibacterial activity of colistin significantly. In addition, the furanone C-30/colistin combination can not only inhibit the formation of bacterial biofilm but also has a better eradication effect on preformed mature biofilms. The result of scanning electron microscopy (SEM) demonstrated that the furanone C-30/colistin combination led to a significant reduction in the number of cells in biofilms. Furthermore, furanone C-30 at 50 μg/ml did not cause any additional toxicity to RAW264.7 cells according to a cytotoxicity assay. In *in vivo* infection experiments, the furanone C-30/colistin combination increased the survival rate of infected Galleria mellonella larvae as well as decreased the microbial load in a mouse thigh infection model. The synergistic effect of the furanone C-30/colistin combination against colistin-resistant GNB is encouraging, and this work may shed light on a new therapeutic approach to combat colistin-resistant pathogens.

**IMPORTANCE** Colistin is among the few antibiotics effective against multidrug-resistant Gram-negative bacteria (GNB) clinical isolates. However, colistin-resistant GNB strains have emerged in recent years. Therefore, the combination of colistin and nonantibacterial drugs has attracted much attention. In this study, the furanone C-30/colistin combination showed good antibacterial and antibiofilm activity *in vitro* and *in vivo*. In addition, increased membrane permeability leads to the synergistic effect of the furanone C-30/colistin combination. Because of the low cytotoxicity of furanone C-30, this combination has good application prospects in clinical anti-infective therapy. This finding might shed light on the discovery of combination therapy for infections caused by colistin-resistant GNB pathogens.

## INTRODUCTION

With the improper and abusive usage of antimicrobials, the appearance of multidrug-resistant (MDR) strains has posed a serious threat to public health worldwide (1). Colistin is a polymyxin antibiotic (polymyxin E) that has emerged as a “last resort” drug for the treatment of MDR Gram-negative bacteria (GNB) and is being widely used in clinical anti-infective treatment. However, colistin-resistant strains have emerged worldwide with different mechanisms, which further aggravates the status quo of antimicrobial drug resistance (2). On this occasion, novel approaches that minimize the emergence of resistant pathogens or increase the efficacy of colistin are desperately needed. As a result, the development of novel antibiotics is not only lagging and challenging (3) but also carries a high risk of failure in the preclinical stage; however, the combination of nontraditional antibiotics and antibiotics as a new treatment scheme has drawn great attention.

Quorum sensing (QS) is a complex population density-based bacterial communication system and is used by many pathogenic bacteria (Staphylococcus aureus, Pseudomonas aeruginosa, and Escherichia coli) to regulate virulence (4, 5). By interrupting or attenuating QS, many natural compounds are used as QS inhibitors to overcome bacterial infections and antibiotic resistance on a variety of pathogens (6–10). Natural furanone compounds show no inhibitory activity against QS (11), but its brominated derivative, furanone C-30, displays an enhanced antagonistic activity against Pseudomonas aeruginosa QS systems (9). The synthetic furanone C-30 was proven to decrease acyl-homoserine lactone-based and autoinducer 2-based QS signaling as well as alleviate the virulence of P. aeruginosa in a mouse pulmonary infection model (9, 12). In addition, these brominated furanones interrupt QS by interacting with transcriptional regulators that suppress virulence (13) without affecting bacterial growth (14).

A biofilm is a community of microorganisms attached to biological and nonliving surfaces and is embedded in an extracellular polymeric substance (EPS) matrix produced by bacteria. Because of its highly adapted ability to survive adverse environmental conditions, it allows pathogens to evade host defenses and immune clearance (15, 16). Furthermore, strong evidence has indicated that biofilm formation is significantly associated with antibiotic resistance by decreasing the penetration of antibiotics, changing the resistance phenotype and microenvironment, etc. (17, 18). Due to adaptive resistance, the major therapeutic strategies against infections are often insufficient to clear biofilm (19). Thus, there is an urgent need to develop novel biofilm-specific therapies.

In the present study, several *in vitro* experiments were performed to evaluate the synergistic antimicrobial and antibiofilm effects of colistin combination with furanone C-30 against colistin-resistant GNB. Moreover, the potential for *in vivo* synergy has been assessed in mice and Galleria mellonella infection models.

## RESULTS

### Colistin-resistant Gram-negative bacteria exhibit multidrug resistance profiles.

The MICs of different isolates against clinical antibiotics are shown in Table S1 in the supplemental material. The result showed that furanone C-30 displayed weak or no antimicrobial activity against colistin-resistant GNB (MIC ≥ 50 μg/ml). In addition, the data in Table 1 showed that most (36/39, 92.3%) of these strains have MDR phenotypes. Among the tested clinical antibiotics, P. aeruginosa showed a higher resistance rate against imipenem than Escherichia coli and Klebsiella pneumoniae. But in general, the drug resistance of E. coli and K. pneumoniae is more severe than P. aeruginosa, as they are resistant to almost all the antibiotics commonly used in the clinic.

**TABLE 1:** Resistance profile of clinical Gram-negative bacterial isolates used in this study

Antibiotic^*a*^	E. coli (*n* = 11)^*b*^			K. pneumoniae (*n* = 17)^*b*^			P. aeruginosa (*n* = 11)^*b*^		
	R (%)	I (%)	S (%)	R (%)	I (%)	S (%)	R (%)	I (%)	S (%)
ATM	90.9	0	9	76.5	0	23.5	54.5	9	36.4
CAZ	100	0	0	88.2	0	11.8	45.5	27.3	27.3
FEP	100	-	0	76.5	-	23.5	36.4	36.4	27.3
IPM	36.4	0	63.6	70.6	0	29.4	72.7	9	18.2
CIP	100	0	0	94.1	0	5.9	54.5	9	36.4
LVX	100	0	0	82.4	5.9	11.8	54.5	18.2	27.3
GEN	81.8	0	18.2	82.4	0	17.6	54.5	27.3	18.2
TOB	72.7	9	18.2	88.2	0	11.8	45.5	27.3	27.3

a ATM, aztreonam; CAZ, ceftazidime; FEP, cefepime; IPM, imipenem; CIP, ciprofloxacin; LVX, levofloxacin; GEN, gentamicin; TOB, tobramycin.

b R, resistant; I, intermediate; S, susceptible.

### Mechanism of colistin resistance.

The colistin resistance genes in the colistin-resistant GNB isolates were analyzed by PCR and sent for sequencing. *mcr-1*, the mobile colistin-resistance gene, was present in all the colistin-resistant E. coli strains. Analysis of the sequences showed that the substitutions in *mgrB*, *PmrB*, and *phoQ* were detected in the majority of colistin-resist K. pneumoniae and P. aeruginosa strains. In addition, *mcr-1* is also prevalent in the four K. pneumoniae strains (Table S2).

### Synergistic activity testing by the checkerboard assay.

According to the checkerboard assay, there was a significant decrease in MIC values for colistin with the addition of furanone C-30. As shown in Table 2, the fractional inhibitory concentration index (FICI) values of colistin/furanone C-30 combinations ranged from 0.04 to 0.5 in almost all the colistin-resistant strains, which showed strong synergism, except for DC5286 (FICI = 0.502, additive effect). None of the strains showed an antagonistic effect.

**TABLE 2:** FICI values for colistin/furanone C-30 combinations against colistin-resistant Gram-negative bacteria

Species	Strains	Monotherapy (μg/ml)		Combination (μg/ml)		FICI	Interpretation
		Colistin	Furanone C-30	Colistin	Furanone C-30		
E. coli	DC90	8	≥200	2	25	0.375	Synergistic
	DC3411	4	100	1	25	0.5	Synergistic
	DC3539	16	100	4	6.25	0.3125	Synergistic
	DC3806	4	≥200	1	25	0.375	Synergistic
	DC3846	8	≥200	2	25	0.375	Synergistic
	DC4887	8	≥200	2	25	0.375	Synergistic
	DC5286	8	100	4	0.2	0.502	Additive
	DC7333	4	≥200	0.5	50	0.375	Synergistic
K. pneumoniae	FK610	32	≥200	4	25	0.25	Synergistic
	FK1342	≥64	≥200	0.5	50	0.2578	Synergistic
	FK1986	16	≥200	0.5	25	0.1875	Synergistic
	FK3810	≥64	100	1	25	0.2656	Synergistic
	FK3994	≥64	≥200	0.5	50	0.2578	Synergistic
	FK6556	32	≥200	2	12.5	0.125	Synergistic
	FK6663	32	100	2	12.5	0.1875	Synergistic
	FK6696	≥64	100	1	25	0.2656	Synergistic
P. aeruginosa	TL1671	16	≥200	2	6.25	0.1406	Synergistic
	TL1736	≥64	≥200	2	3.125	0.047	Synergistic
	TL1744	32	≥200	0.5	6.25	0.047	Synergistic
	TL2314	8	≥200	0.5	12.5	0.125	Synergistic
	TL2917	16	≥200	2	6.25	0.156	Synergistic
	TL2967	4	≥200	1	25	0.375	Synergistic
	TL3008	≥64	≥200	2	6.25	0.0625	Synergistic
	TL3086	≥64	≥200	2	6.25	0.0625	Synergistic

### Time-kill assay.

**FIG 1:**
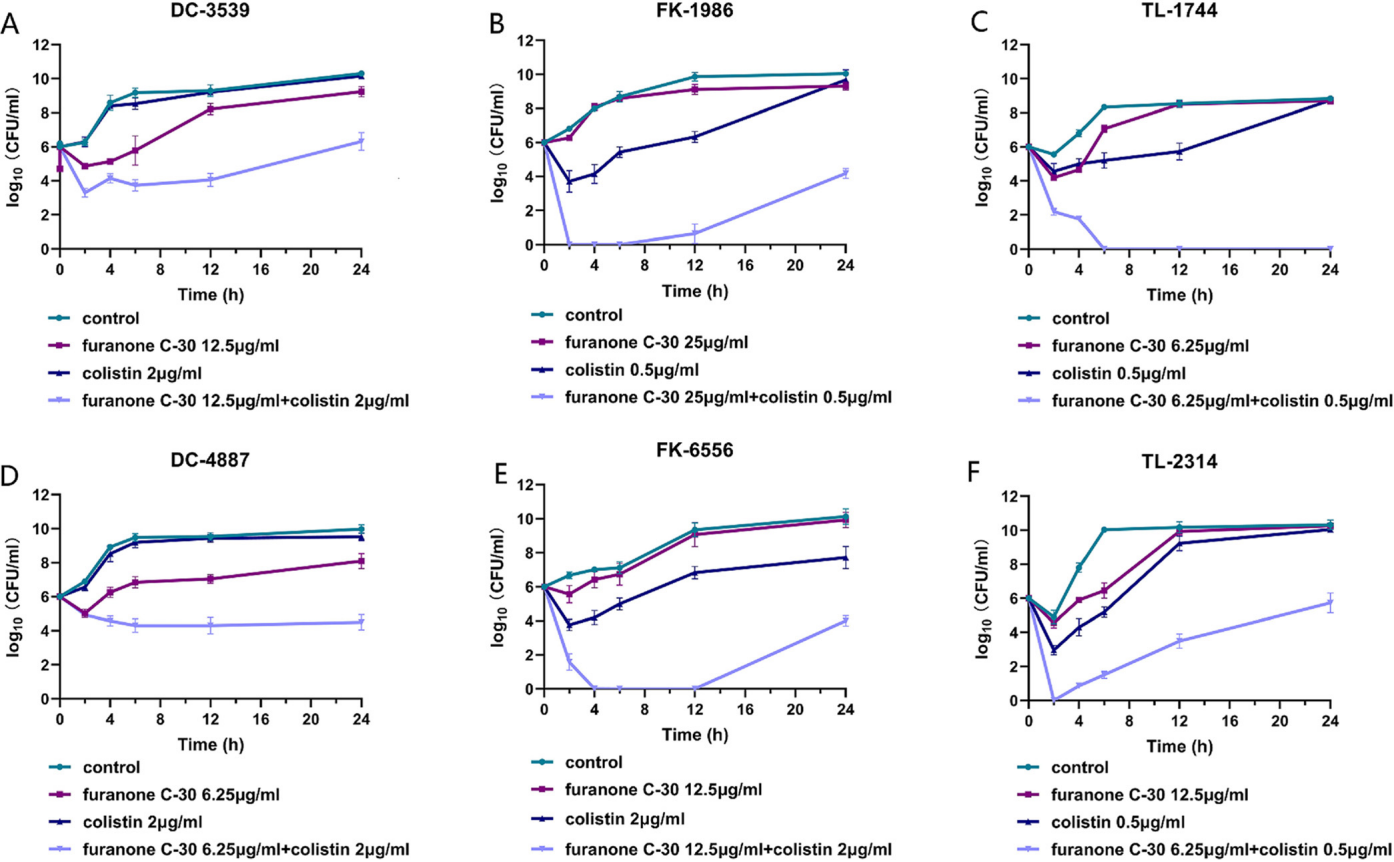
Time-killing curves of colistin and furanone C-30 alone or in combination against colistin-resistant GNB. (A, B) Colistin-resistant E. coli. (C, D) Colistin-resistant Klebsiella pneumonia. (E, F) Colistin-resistant Pseudomonas aeruginosa. The experiments were performed three times. Data are expressed as mean ± standard deviation.

Time-kill curves against 6 colistin-resistant isolates (2 P. aeruginosa, 2 E. coli, and 2 K. pneumoniae strains) are shown in Fig. 1. Bacteria exposed to each drug alone displayed a killing curve comparable to the control cultures not exposed to drugs. In contrast, the combination of furanone C-30 and colistin resulted in more rapid killing, and all strains treated with the combination of furanone C-30 and colistin demonstrated a dramatic decrease in viable cells greater than 2 log_10_ (CFU/ml)-fold by 24 h compared to that observed with either agent alone. In summary, the bactericidal activity of colistin was significantly enhanced when in combination with furanone C-30.

### The antibiofilm activity of colistin/furanone C-30 combinations.

**FIG 2:**
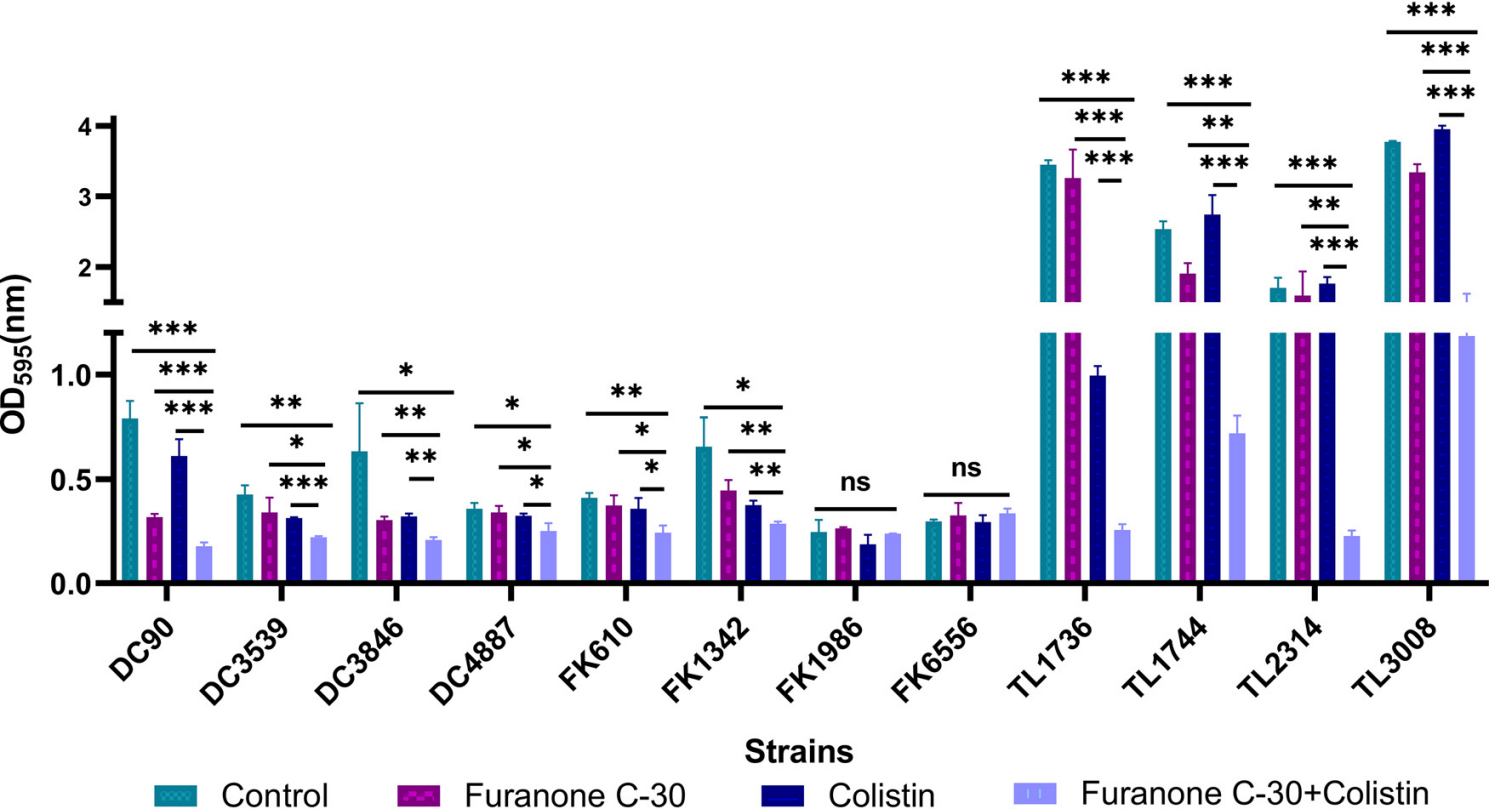
Biofilm inhibitory effects of colistin combined with furanone C-30 on colistin-resistant GNB. Data were analyzed by Student’s *t* test; ns, not statistically significant; *, *P < *0.05; **, *P < *0.01; ***, *P < *0.001. The experiments were performed three times. Data are expressed as mean ± standard deviation; OD_595_, optical density at 595 nm.

**FIG 3:**
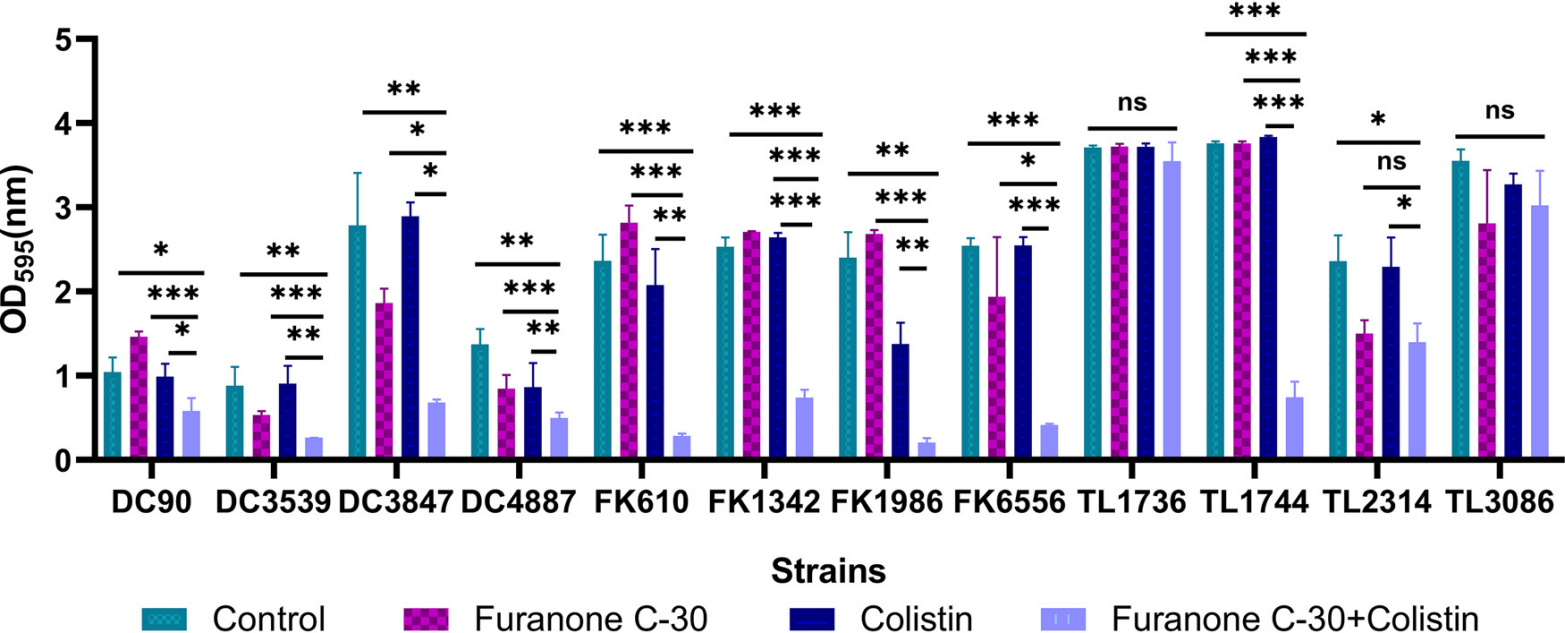
Biofilm eradication effects of colistin combined with furanone C-30 on colistin-resistant GNB. Data were analyzed by Student’s *t* test; ns, not statistically significant; *, *P < *0.05; **, *P < *0.01; ***, *P < *0.001. The experiments were performed three times. Data are expressed as mean ± standard deviation.

We investigated the ability of furanone C-30 and colistin, singly or in combination, to inhibit the formation of biofilms and eradicate the preformed mature biofilms of colistin-resistant GNB strains. As shown in Fig. 2, with the synergistic concentration obtained from the checkboard method, biofilm formation of each strain was decreased significantly compared to the cells treated with furanone C-30 or colistin alone in almost all the GNB strains (*P < *0.05). In addition, colistin/furanone C-30 combinations showed obvious activity on preformed mature biofilm (Fig. 3).

### Scanning electron microscopy.

**FIG 4:**
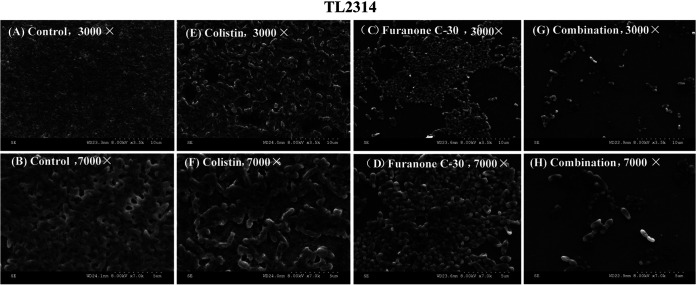
Scanning electron microscopy (SEM) of the effects of the furanone C-30 and colistin combination treatment on bacterial number and biofilm formation of colistin-resistant Pseudomonas aeruginosa TL-2314. (A) LB broth control at ×3,000 magnification. (B) LB broth control at ×7,000 magnification. (C) Colistin alone at ×3,000 magnification. (D) Colistin alone at ×7,000 magnification. (E) Furanone C-30 alone at ×3,000 magnification. (F) Furanone C-30 alone at ×7,000 magnification. (G) Furanone C-30/colistin combination at ×3,000 magnification. (H) Furanone C-30/colistin combination at ×7,000 magnification.

To further characterize the inhibitory effect of the furanone C-30/colistin combination on biofilms, scanning electron microscopy (SEM) was performed (Fig. 4). SEM analysis revealed that untreated P. aeruginosa cells exhibited a thick biofilm with regular and stable cell morphology. Biofilms treated with furanone C-30 (12.5 μg/ml) and colistin (1 μg/ml) alone also formed abundant biofilms with complete morphology and dense arrangement. However, samples treated with furanone C-30 combined with colistin showed a significant decrease in cells. In addition, at ×7,000 magnification, bacterial cells treated with furanone C-30/colistin combinations showed abnormalities in their cell envelopes, including the appearance of vesicles, bulges, wrinkles, and partial destruction.

### *In vivo* treatment verification.

**FIG 5:**
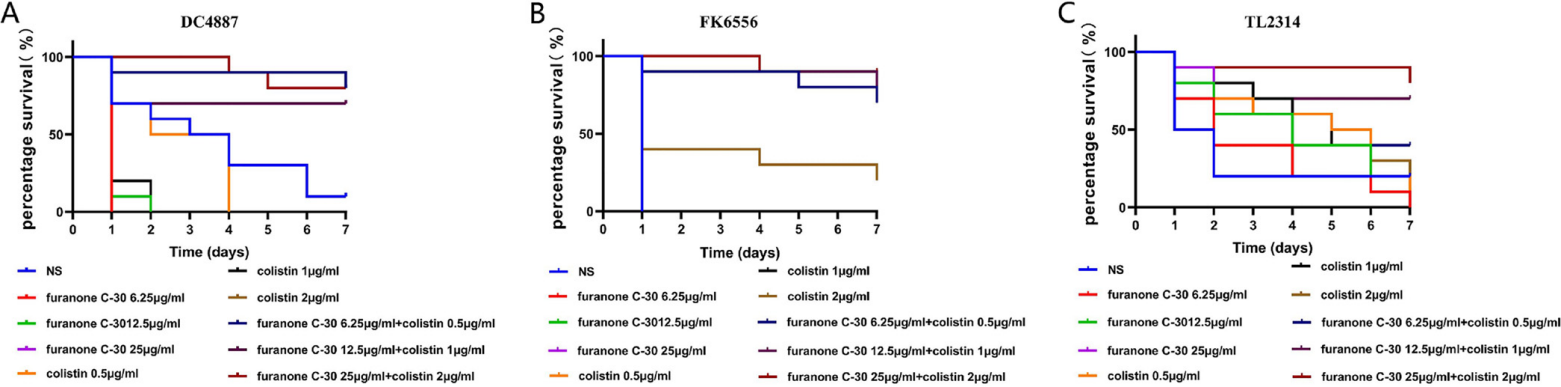
Survival rate of G. mellonella after 7 days of monotherapy or combination therapy using different dosing regimens against colistin-resistant P. aeruginosa TL-2314 and K. pneumoniae FK6556. (A) Colistin-resistant E. coli DC4887. (B) Colistin-resistant Klebsiella pneumonia FK6556. (C) Colistin-resistant Pseudomonas aeruginosa TL2314; NS, not statistically significant.

To ascertain the *in vivo* therapeutic effect of the colistin/furanone C-30 combination against colistin-resistant GNB strains, a G. mellonella survival experiment was developed. According to the results shown in Fig. 5, almost all the G. mellonella administered with phosphate-buffered saline (PBS) and dimethyl sulfoxide (DMSO) were dead after 168 h, and the survival rate of G. mellonella with monotherapy was significantly lower than that observed with the combination therapy (*P < *0.05).

**FIG 6:**
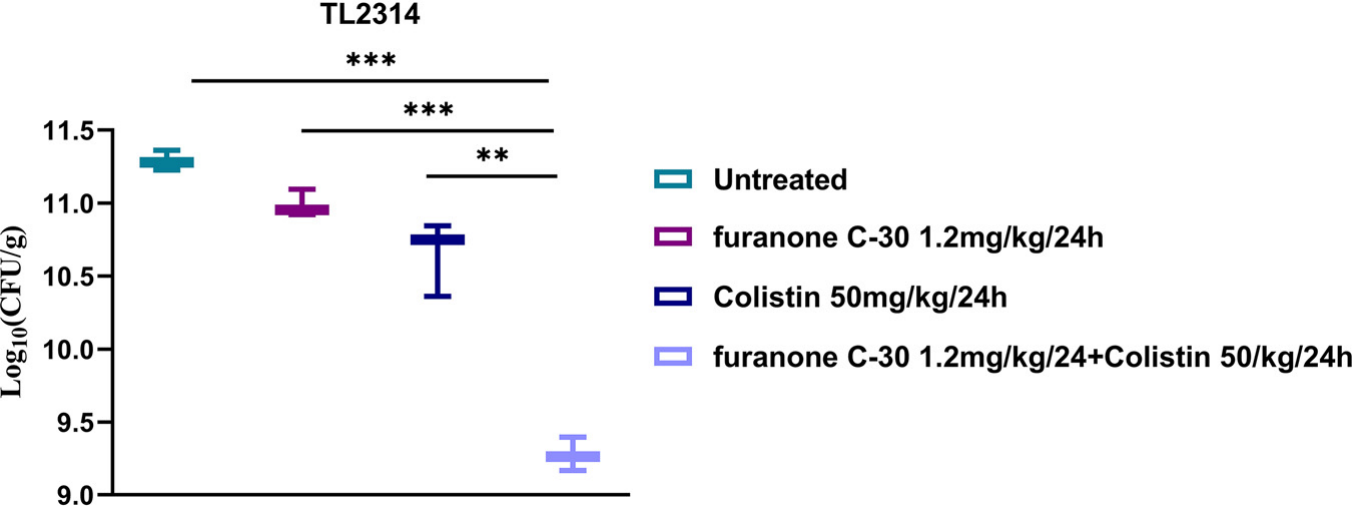
Log_10_ changes in mouse thigh muscles (Δlog_10_ CFU/thigh) after 24 h of monotherapy (furanone C-30 at 1.2 mg/kg and colistin at 5 mg/kg) or combination therapy (1.2 mg/kg furanone C-30 + 5 mg/kg colistin) using different dosing regimens against colistin-resistant P. aeruginosa TL-2314. Data were analyzed by Student’s *t* test; **, *P < *0.01; ***, *P < *0.001. The results represent means ± standard deviations, and the data are representative of at least 3 independent experiments.

At the same time, the *in vivo* therapeutic effect of the colistin/furanone C-30 combination was further assessed by the neutropenic mouse thigh infection model. Data from colistin and furanone C-30 administered as monotherapies or in combination are shown in Fig. 6. Furanone C-30 at 1.2 mg/kg and colistin at 5 mg/kg lightly inhibited P. aeruginosa isolate TL2314 after injection for 24 h. Additionally, colistin combined with furanone C-30 (1.38 log_10_ [CFU] reduction) showed higher efficacies than when used alone. The *in vivo* experiments indicated that treatment with the combination of the two drugs to colistin-resistant P. aeruginosa TL-2314 has a significant synergistic antibacterial effect.

### *In vitro* evaluation of cytotoxicity.

**FIG 7:**
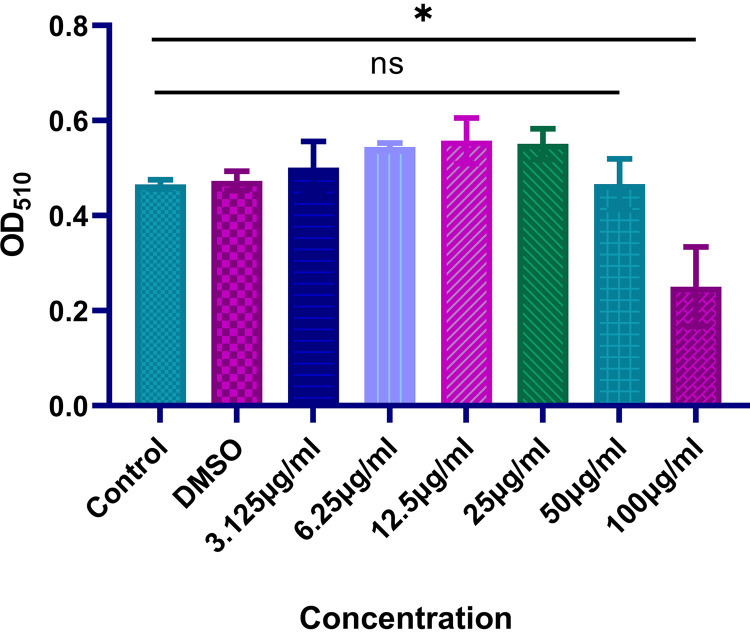
Cytotoxic effect of furanone C-30 with different concentrations and 2.5% DMSO against the RAW 264.7 murine macrophage cell line (absorbance values at 490 nm). Data were analyzed by Student’s *t* test; ns, not statistically significant; *, *P < *0.05. The results are shown as the mean and standard deviation of three independent experiments; DMSO, dimethyl sulfoxide.

In this work, we investigated any potential toxic effects of furanone C-30 on RAW264.7 cells. The result showed that the concentration of 50 μg/ml of furanone C-30 did not cause any additional toxicity to the RAW264.7 cell line compared with the control group treated with DMSO or water (Fig. 7). This result indicated that the combination regimen used in this study can be safely used *in vivo*.

### Mechanism of synergy action studies.

**FIG 8:**
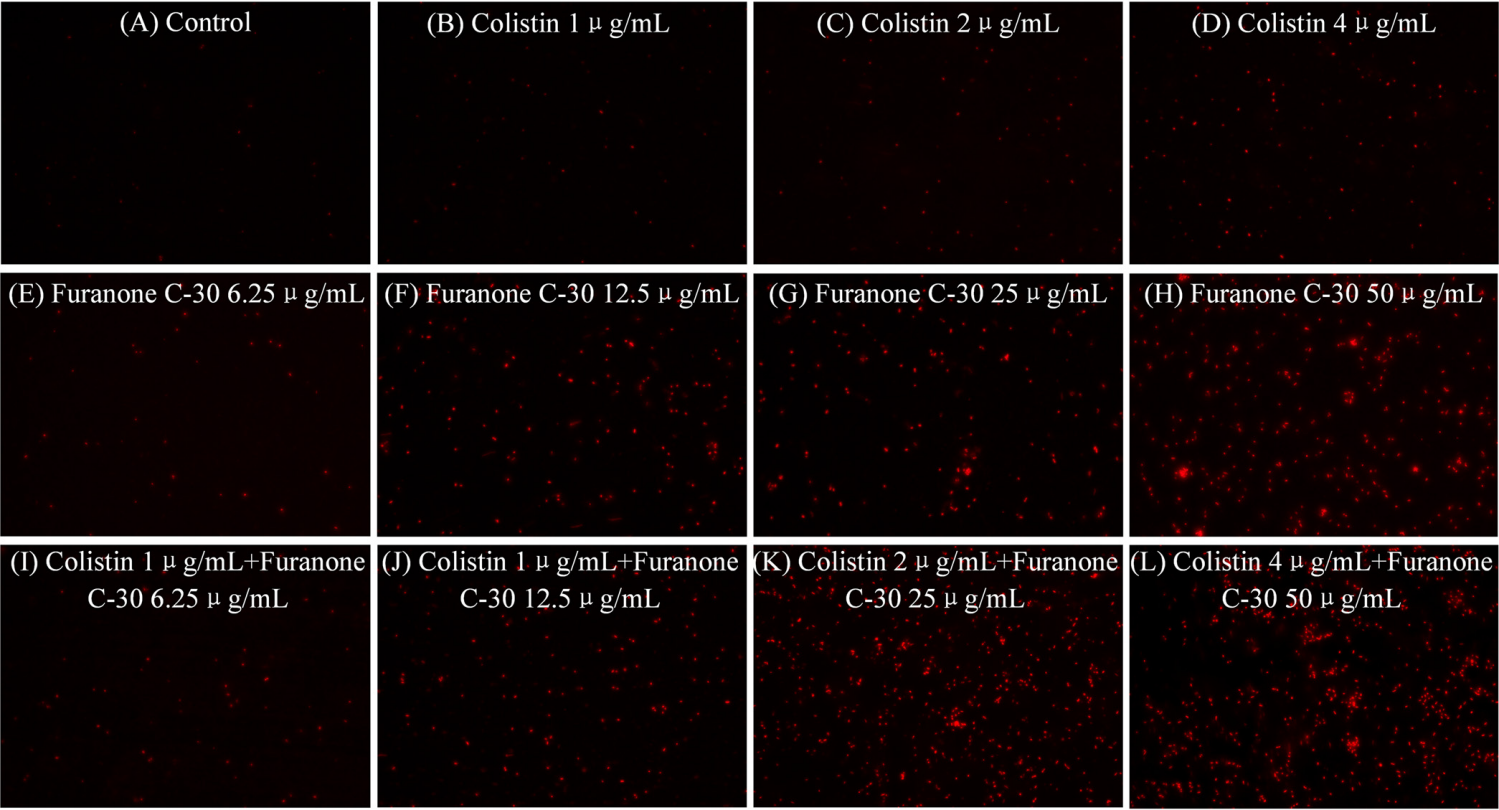
Fluorescence microscopy imaging of exponential-phase P. aeruginosa TL-2314, which were treated with furanone C-30 and colistin alone or in combination and incubated with 50 μg/ml PI for 10 min before imaging. (A) LB broth control. (B to D) Cells treated with colistin at 1 μg/ml (B), 2 μg/ml (C), and 4 μg/ml (D). (E to H) Cells treated with furanone C-30 at 6.25 (E), 12.5 (F), 25 (G), and 50 μg/ml (H). (I to L) Cells exposed to a combination of furanone C-30 and colistin.

**FIG 9:**
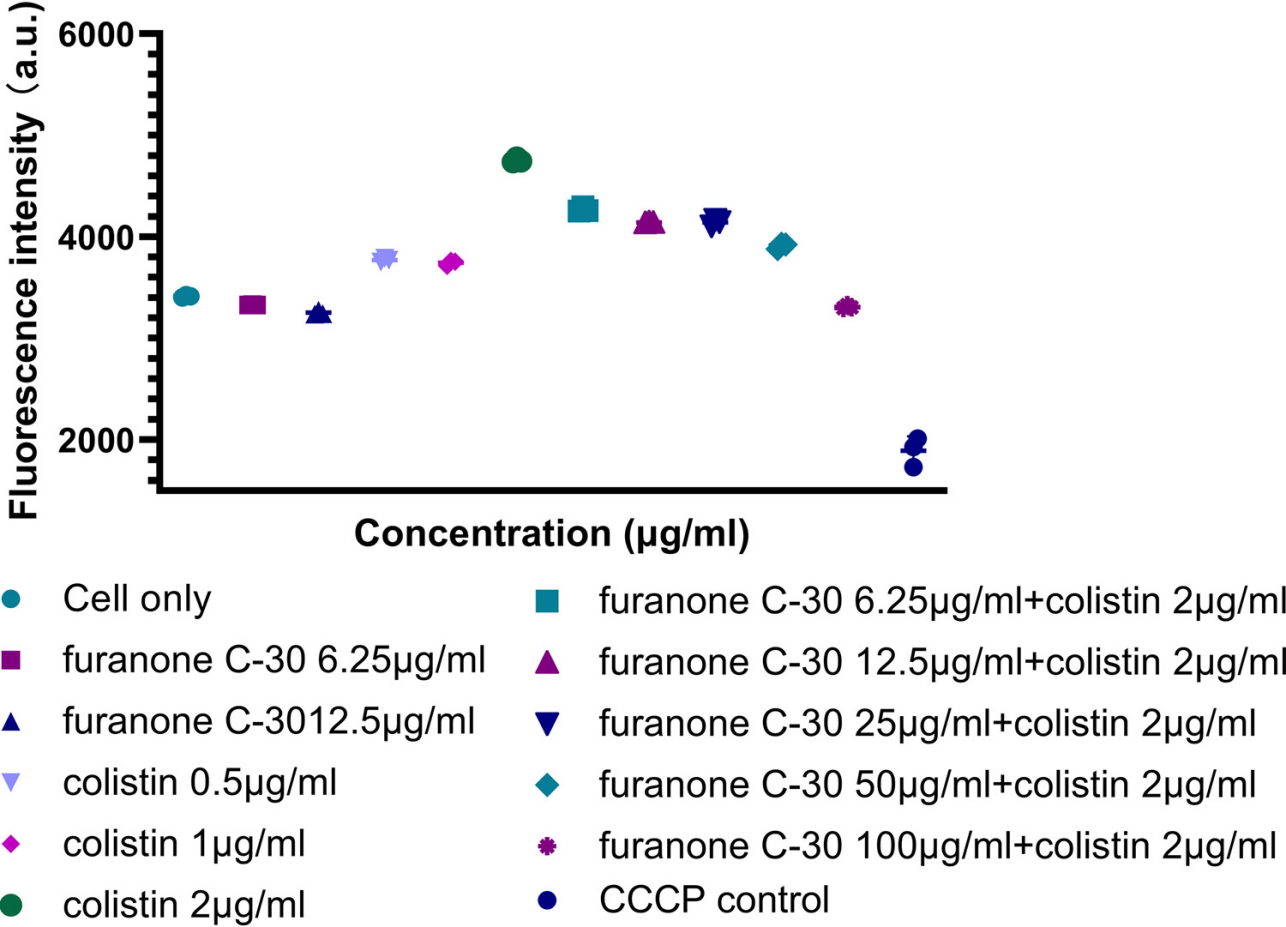
Membrane potential measurements of P. aeruginosa TL-2314 after treatment with furanone C-30 and/or colistin. Cells were treated with furanone C-30 and/or colistin at the indicated concentrations for 30 min, after which DiOC_2_(3) was added, and the fluorescence was monitored. The membrane potential was disrupted by addition of the proton ionophore carbonyl cyanide *m*-chlorophenylhydrazone (CCCP); a.u., arbitrary units.

We evaluated the cell membrane permeability of TL2314 using propidium iodide (PI). As revealed by fluorescence microscopy analysis (Fig. 8), colistin had little effect on cell membrane permeability. However, preincubation of cells with furanone C-30 resulted in a concentration-dependent increase in fluorescence intensity due to PI uptake and DNA binding, which indicated that the integrity of the cell membrane gradually decreased. Additionally, accompanying the severe membrane injury, dissipation of membrane potential was observed by a membrane potential assay (Fig. 9). When cells were preincubated with colistin at 1 μg/ml or 2 μg/ml, the outer membrane of P. aeruginosa was permeabilized so that greater quantities of the membrane potential probe 3,3′-diethyloxacarbocyanine iodide [DiOC2(3)] could gain entry into cells with enhancing membrane potential. As expected, preincubation of cells with the furanone C-30/colistin combination resulted in a furanone C-30 concentration-dependent deenergized membrane potential. Taken together, the results indicated that the furanone C-30-mediated membrane disruption mechanism may enhance the bactericidal effect of colistin.

## DISCUSSION

With the rapidly dwindling number of therapeutic options, MDR allocated to Gram-negative infection has become a current problem requiring urgent attention (20). Under this circumstance, colistin has been proposed as a last-line antibiotic for the treatment of MDR Gram-negative pathogen infections. However, colistin resistance has been increasingly reported among Gram-negative clinical pathogens (21).

The ability of furanone C-30 to inhibit the QS of P. aeruginosa has been widely reported previously (5, 22). Unlike classic antibiotics, which often interfere with the biological metabolism of bacteria involved in basal life processes, furanones might comprise a natural chemical defense system developed through the course of evolution to target and inactivate receptors of bacterial communication systems that, in turn, control virulence factor production, surface colonization, and biofilm formation (9). Recently, the combination of furanone C-30 with tobramycin against P. aeruginosa has been shown to enhance antimicrobial activity (23). However, whether furanone C-30 could reverse colistin resistance remains to be further elucidated.

In this work, we examined the *in vitro* and *in vivo* activity of furanone C-30 in combination with subinhibitory concentrations of colistin to develop a new treatment method against colistin-resistant GNB pathogens. The results of antimicrobial susceptibility assays indicated that 92.3% (36/39) of these strains showed multidrug-resistant phenotypes. *In vitro* antibacterial activity by the checkerboard method and time-kill curves showed that the furanone C-30/colistin combination posed a significant synergistic effect in most tested strains (except for DC5286 [additive effect]).

In addition, the combination of furanone C-30 and colistin can not only inhibit the formation of bacterial biofilms but also has a better eradicative effect on preformed mature biofilms. SEM results demonstrated that compared with the alone-drug group, the furanone C-30 and colistin combination showed a significant reduction in the number of cells in the biofilm. For P. aeruginosa, it has been demonstrated that the ability to form biofilms is affected by QS (24). Whether furanone C-30 also has an inhibitory effect on the quorum sensing system of E. coli and K. pneumoniae is unknown. However, although the furanone C-30/colistin combination showed a conspicuous effect on inhibitory biofilm formation and eradication of preformed mature biofilms, furanone C-30 alone had no obvious inhibition on the biofilms of P. aeruginosa. We speculate that this may be due to the furanone concentration we used and that it did not reach the required concentration to inhibit the QS system of P. aeruginosa.

To further evaluate the *in vivo* therapeutic effect of the combined regimen, we constructed an infection model in mice and G. mellonella. The results showed that the furanone C-30/colistin combination can significantly reduce the number of bacteria in mice and improved the survival rate of G. mellonella compared to the control group. In addition, the safety of furanone C-30 has been verified by a cytotoxicity test, and the results show that furanone C-30 has low toxicity and can be used safely in humans.

As an inhibitor of the QS system, we envision that furanone C-30 can directly target the virulence of planktonic and biofilm-producing bacteria and is promising as an early prophylactic treatment of bacterial infection. These so-called “antivirulence” treatments should exert weaker selection for resistance than classical antibiotics because they simply disable virulence factors but are not supposed to affect pathogen viability (25–27). Furthermore, these data strongly support that the furanone C-30/colistin combination can reverse the colistin-resistant phenotype and antibiofilm activity effectively. Combining antivirulence compounds with antibiotics is a potentially promising strategy for both virulence suppression and effective pathogen removal.

## CONCLUSION

Overall, this is the first report of synergistic activity of colistin in combination with furanone C-30 in Gram-negative bacteria, and the synergistic effect of the furanone C-30/colistin combination is encouraging both *in vitro* and *in vivo*. The results of this study provide a useful platform for drug combination optimization and suggest a reduced likelihood of failure of drug combination during therapy.

## MATERIALS AND METHODS

### Antibiotics and solvents.

Furanone C-30 was purchased from MedChemExpress (MCE) Co., Ltd. (NJ, USA) and was dissolved in 1% (vol/vol) dimethyl sulfoxide (DMSO) (Sigma-Aldrich, Saint Louis, MO, USA). All antibiotics used in this study, including colistin, aztreonam, ceftazidime, cefepime, imipenem, ciprofloxacin, levofloxacin, gentamicin, tobramycin, and amikacin, were purchased from Wenzhou Kangtai Biological Technology Co., Ltd. (Zhejiang, China). Solvents and diluents for the preparation of antibiotics complied with the latest guidelines published by the Clinical and Laboratory Standards Institute (CLSI 2020) (28).

### Bacterial isolates and growth conditions.

A total of 39 nonduplicated Gram-negative clinical colistin-resistant isolates were recovered from the First Affiliated Hospital of Wenzhou Medical University in China from 2015 to 2017, including colistin-resistant P. aeruginosa (*n* = 11), E. coli (*n* = 11), and K. pneumoniae (*n* = 17). All species identification was performed by using matrix-assisted laser desorption ionization–time of flight mass spectrometry (MALDI-TOF MS; bioMérieux, France). All bacteria were stored at −80°C in Luria-Bertani (LB) broth medium (Oxoid, UK) with 30% glycerol for further use. P. aeruginosa ATCC 27853 and E. coli ATCC 25922 served as the quality controls, which were purchased from the National Center of the Clinical Laboratory (NCCL). All of the investigation protocols in this study were approved by the Ethics Committee of the First Affiliated Hospital of Wenzhou Medical University.

### Determination of antimicrobial resistance profiles.

The MICs of antibiotics and furanone C-30 were determined against 39 clinical GNB isolates by microdilution assay as described previously (29). Briefly, the 96-well flat-bottom microtiter plates containing a series of diluted antibiotics were prepared. Each well of the plate was inoculated with 100 μl of the cell suspension at 37°C for 16 to 18 h. The breakpoint of antibiotics was interpreted accordingly to the CLSI. Each MIC test against all isolates was performed in duplicate.

### PCR and sequencing.

We have explored the resistance mechanism of some colistin-resistant GNB strains previously (30–33). Whole-cell DNA of colistin-resistant isolates was extracted using the Bio-Spin bacterial genomic DNA extraction kit (BioFlux, Tokyo, Japan) according to the manufacturer’s instructions. The colistin resistance-related genes (*mcr-1*, *mgrB*, *pmrA*, *pmrB*, *phoP*, *phoQ*, *parR*, *parS*, *cprR*, and *cprS*) were detected by PCR using the primers and conditions described in Table S3 in the supplemental material. The positive PCR products were sent to Shanghai Majorbio Bio-Pharm Technology Co. (Shanghai, China) for sequencing. The sequences were compared with the standard strain deposited in the NCBI database using BLASTn and BLASTx programs (http://blast.ncbi.nlm.nih.gov/Blast.cgi). The online PROVEAN platform (http://provean.jcvi.org/seq_submit.php) was used to predict the alterations in biological functions of the proteins.

### Checkerboard assays.

Twenty-four GNB isolates were randomly selected (8 E. coli, 8 K. pneumoniae, and 8 P. aeruginosa) for the checkerboard method according to the standard checkerboard assay with some modifications (34). Briefly, the two drugs were diluted with cation-adjusted Mueller-Hinton broth (CAMHB) into a series of concentrations based on the MIC for each tested strain. The alone bacterial colony grown overnight was diluted to a 0.5 McFarland standard in sterile saline, followed by a 1:100 subsequent dilution in CAMHB. The final bacteria concentration of each well was approximately 7.5 × 10^5^ CFU/ml. Results were observed after incubation at 37°C for 16 to 20 h. The experiments were performed in triplicate.

Synergy was evaluated by calculating the fractional inhibitory concentration index (FICI). The FIC_A_ was obtained by dividing the concentration of drug A when used in combination (C_A_) by the MICs of the drug when used alone (MIC_A_). Similarly, FIC_B_ was obtained by dividing the concentration of drug B when used in combination (C_B_) by the MIC of the drug when used alone (MIC_B_) (FICI = FIC_A_ + FIC_B_ = [C_A_/MIC_A_] + [C_B_/MIC_B_]; interactions were interpreted as follows: synergy for FICI ≤ 0.5, additive for 0.5 < FICI ≤ 1, irrelevant for 1 < FICI ≤ 2, and antagonistic for FICI > 2) (35).

### Time-kill assay.

The colistin-resistant P. aeruginosa (*n* = 2), E. coli (*n* = 2), and K. pneumoniae (*n* = 2) strains we used for time-kill assays were based on the results of the checkerboard assay. The time-kill assay was conducted as described previously with modifications (36, 37). Briefly, bacteria were exposed to furanone C-30 and colistin alone or in combinations at 1 × 10^6^ CFU/ml. Tubes containing LB alone served as the negative control. The bacterial suspensions were incubated at 37°C with moderate shaking for 0, 2, 4, 6,12, and 24 h. The CFU was counted after overnight incubation at 37°C. Bactericidal activity was defined as a ≥3 log_10_ decrease in CFU/ml by 24 h, and synergistic activity was defined as a ≥2 log_10_ decrease in CFU/ml at 24 h by the two-drug combination compared with either drug alone (36). Then, we calculated the means ± standard deviation (SD) of viable CFU and plotted it on a semilogarithmic graph. The assay was performed in triplicate for each strain.

### Biofilm formation inhibition assays.

Biofilm formation inhibition assays were performed as previously described with some modifications (38). The study was carried out with 12 colistin-resistant strains (4 P. aeruginosa, 4 E. coli, and 4 K. pneumoniae). The single colony on the blood plate was selected and grown overnight in 3 ml of fresh LB broth medium at 37°C with moderate shaking. Then, the culture was adjusted to a 0.5 McFarland standard with sterile saline and 1:100 dilution in LB broth before being dispensed in the 96-well microtiter plate with 12.5 μg/ml of furanone C-30 and 1 μg/ml colistin alone or in combination. After incubation at 37°C for 24 h, planktonic and nonadherent cells were removed by washing twice with 200 μl of 1× PBS (Sigma-Aldrich, Milan, Italy), and the plate was inverted to dry at room temperature. Following this, 150 μl of 1% crystal violet (CV) solution (Beijing Solarbio Biotechnology Co., Ltd., China) was added to each well for 15 min. After staining, CV was removed, and the wells were washed 3 times with 1× PBS. The bound CV was then solubilized with 150 μl of ethanol-acetone (96:5 vol/vol). The absorbance was read at 595 nm on a microplate reader (Multiskan FC). The experiments were performed in triplicate and repeated three times.

### Preformed mature biofilm eradication assays.

The potential synergism of colistin/furanone C-30 combinations against preformed biofilms was investigated using a standardized *in vitro* biofilm model (39). In brief, overnight cultured colistin-resistant P. aeruginosa (*n* = 4), E. coli (*n* = 4), and K. pneumoniae (*n* = 4) strains in 3 ml of fresh LB broth were adjusted to a 0.5 McFarland standard by sterile saline and followed by1:100 dilution in LB broth. LB broth (100 μl) and an aliquot of each sample were then transferred to a 96-well microtiter plate and incubated at 37°C for 24 h. Preformed biofilms were then exposed to 8 μg/ml of colistin and 25 μg/ml of furanone C-30, alone or in combination, for 24 h (static conditions, 37°C). Loosely adherent bacteria were removed by washing twice with 200 μl of PBS. After that, 100 μl of PBS was added to the wells along with 10 μl of 3-(4,5-dimethyl-2-thiazolyl)-2,5-diphenyl-2H-tetrazolium bromide (MTT) solution. The plate was incubated for 2 h in the dark, 110 μl of solvent solution (DMSO) was added, and the plate was incubated for 15 min at ambient temperature with gentle agitation. The absorbance was read at 570 nm in a microplate reader. Data were obtained from at least two independent experiments with at least three replicates per condition.

### Scanning electron microscopy.

For the scanning electron microscopy (SEM) experiments, the overnight-grown culture of TL2314 was adjusted to a 0.5 McFarland standard by sterile normal saline, and sterile coverslips were placed in each well of a 6-well plate (NEST Biotechnology Co., Ltd., China). Diluted culture (100 μl) was aliquoted into each well of the 6-well plates with 1,900 μl furanone (12.5 μg/ml) and colistin (1 μg/ml) singly or in combination and incubated at 37°C under static conditions for 18 to 24 h. The contents were aspirated and washed with 200 μl of 1 × PBS. Biofilm samples were fixed using 2.5% glutaraldehyde fixation solution in a new 6-well plate at 4°C for 4 h, and the samples were dehydrated by increasing concentrations of ethanol (20%, 40%, 70%, 90%, 95%, and 100% [vol/vol], 2 min each). All samples were then allowed to dry at 37°C for 15 min and visualized by SEM (S-3000N, Japan).

### *In vivo* evaluation of synergy in a Galleria mellonella infection model.

The efficacy of colistin alone and combined with furanone C-30 in G. mellonella infected with Gram-negative bacteria was evaluated by survival assays, as described previously with modifications (40, 41). Overnight cultures of P. aeruginosa (TL2314), E. coli (DC4887), and K. pneumoniae (FK6556) strains were washed with phosphate-buffered saline (PBS) and further adjusted with PBS to concentrations of 1 × 10^5^ CFU/ml. Insects between 250 and 350 mg were selected. The insects injected with PBS were used as controls. Bacteria solution (10 μl) was injected into the rear left proleg of G. mellonella by using a microinjector and treated with the test drug alone (furanone C-30 6.25, 12.5, 25 μg/ml × 7; colistin 0.5, 1, 2 μg/ml × 7) or in combination (furanone C-30 6.25 μg/ml + colistin 0.5 μg/ml, furanone C-30 12.5 μg/ml + colistin 1 μg/ml, furanone C-30 25 μg/ml + colistin 2 μg/ml × 7) of 7 MICs after 2 h of infection. Insects were placed at 37°C, and the survival rates of G. mellonella were recorded after 6, 12, 24, 48, 72, 96, 120, 144, and 168 h. All experiments were done in triplicate. Larvae were considered dead when they repeatedly failed to respond to physical stimuli. The primary outcome for the insect model was the rapidity and extent of mortality of G. mellonella assessed by Kaplan-Meier analysis and log rank test.

### *In vivo* evaluation of synergy in the mouse infection model.

The neutropenic mouse thigh infection model was constructed for *in vivo* studies. Specific pathogen-free (SPF) female ICR mice, 5 to 6 weeks old (Charles River, Hangzhou, China), were used in this experiment. Mice were maintained following the National Standards for Laboratory Animals of China (GB 14925–2010). All animal studies were approved by the Zhejiang Association for Science and Technology (ID: SYXK [Zhejiang] 2018-0017) and conducted in accordance with Wenzhou Laboratory Animal Welfare and Ethics guidelines.

In short, neutropenia (neutrophils ≤ 100/mm^3^) was induced by injecting cyclophosphamide (Yuanye Biotechnology Co., Ltd., Shanghai, China) intraperitoneally at 4 days (150 mg/kg) and 1 day (100 mg/kg) before thigh infection. A colistin-resistant strain (TL2314) was selected according to a previous study, and 100 μl of exponentially growing bacterial suspension of 1.5 × 10^7^ CFU/ml was injected into each posterior thigh muscle. Two hours after bacterial inoculation, colistin was administered intraperitoneally at 5 mg/kg every 24 h as a monotherapy or in combination with furanone C-30 (2 mg/kg every 24 h) (9). The mice were euthanized after 24 h of therapy as were untreated control mice. Bacterial burden was quantified by CFU counts from posterior thigh homogenates. Groups of three mice (6 thigh infections) were included in each dosing regimen.

### *In vitro* cytotoxicity assays.

The cytotoxicity test was conducted on RAW264.7 cells (ATCC, Manassas, VA). RAW264.7 cells were cultured in Dulbecco’s Modified Eagle Medium (DMEM) augmented with 10% heat-inactivated fetal bovine serum (FBS) and incubated in a 5% CO_2_ incubator at 37°C until confluent. Confluent cells were harvested by trypsin. About 100 μl of cell suspension with 1 × 10^5^ cells was plated into each well of the 96-well microplates. After being incubated for 24 h, the medium was added with 10 μl of serial concentrations of furanone C-30 (3.125, 6.25, 12.5, 25, 50, and 100 μg/ml), and the plate was then incubated for 12 h. FBS containing the DMSO (solvents of furanone C-30) served as the negative control. After incubation, 10 μl of CCK-8 (Dojindo Laboratories, Japan) was added to each well and incubated in the dark at room temperature for 1 h. The absorbance was recorded using a microplate reader at 450 nm.

### Propidium iodide staining.

Cell membrane permeability was examined as described previously with modifications (42). Exponential-phase cells of TL2314 were treated with the test drug alone (furanone C-30 6.25, 12.5, 25, and 50 μg/ml; colistin 1, 2, and 4 μg/ml) or in combination for 2 h and incubated at room temperature in propidium iodide (PI) (50 μg/ml) for 10 min. The fluorescence was examined on a fluorescence microscope (Nikon, Japan) at 561 nm, and images were recorded.

### Membrane potential assay.

To examine the perturbation of the cell membrane of TL2314, the membrane potential assay was conducted as described elsewhere, with some modifications (43, 44). For this assay, furanone C-30 was used at 6.25, 12.5, 25, 50, or 100 μg/ml, while colistin was used at 0.5, 1, or 2 μg/ml. Cells were treated with the individual compounds or their combinations at ambient temperature for 30 min with stirring before and during the assay. Ten microliters of 500 μM CCCP was added to the depolarized control sample. The fluorescent membrane potential probe 3,3′-diethyloxacarbocyanine iodide [DiOC_2_(3)] (MaoKang Biotechnology, Inc., Shanghai, China) at a final concentration of 30 μM was added and incubated at room temperature for 30 min. The membrane potential of the cells was measured by a multifunctional microplate reader (BioTek) at an excitation of 486 nm and emission of 620 nm. All assays were performed in triplicate.

### Statistical analysis.

A chi-square test and Fisher’s exact test were used to compare categorical variables. A Student’s *t* test and Mann-Whitney U test were used to compare continuous variables with and without normal distribution, respectively. Statistical significance was tested by paired two-tailed *t* test and one-way analysis of variant (ANOVA), and a *P* value of <0.05 was considered significant; *, *P < *0.05, **, *P < *0.01, and ***, *P < *0.001 for all analyses. Data were analyzed by SPSS version 22.0 (SPSS Inc., Chicago, IL, USA).

### Data availability.

All data generated or analyzed during this study are included in this published article.
