# Supplementary material for: Combining Colistin with Furanone C-30 Rescues Colistin Resistance of Gram-Negative Bacteria in Vitro and in Vivo
Source: Microbiol Spectr. 2021 Nov 3;9(3):e01231-21. doi: 10.1128/Spectrum.01231-21 (PMC8567244; doi:10.1128/Spectrum.01231-21)
Supplement: SUPPLEMENTAL FILE 1 — Supplemental material. Download Spectrum.01231-21-s0001.pdf, PDF file, 0.3 MB [file spectrum.01231-21-s0001.pdf]

Table S1 The minimum inhibitory concentrations (MICs) of commonly used clinical antibiotics and furanone C-30 against colistin-resistant GNB.

**Species**	**Strains[a]**	**Antibiotics[b]**	**ATM**	**CAZ**	**FEP**	**IPM**	**CIP**	**LVX**	**GEN**	**TOB**	**COL**	**Furanone C-30**
		**Breakpoints (S-R)**	8-32	8-32	8-32	2-8	0.5-2	1-4	4-16	4-16	2-4	
P. aeruginosa	TL1671		8	4	8	2	0.25	1	2	1	**16**	**≥200**
	**TL1722**		0.5	**128**	**64**	1	≥256	≥256	≥256	**128**	**≥64**	**≥200**
	**TL1736**		4	4	2	**16**	1	1	**32**	8	**≥64**	**≥200**
	**TL1744**		**32**	**32**	16	**16**	**32**	**8**	≥256	**32**	**32**	**≥200**
	**TL2206**		≥256	≥256	≥256	≥256	**2**	**4**	4	≥256	**4**	**≥200**
	**TL2294**	**MIC (μg/mL)**	≥256	≥256	≥256	≥256	≥256	**64**	**32**	**64**	**4**	**50**
	TL2314		16	**32**	16	4	0.5	2	8	2	**8**	**≥200**
	**TL2917**		**32**	16	16	**16**	0.25	2	8	8	**16**	**≥200**
	**TL2967**		**128**	16	**32**	**16**	**8**	**16**	8	8	**4**	**≥200**
	**TL3008**		4	2	4	**16**	0.5	1	**16**	4	**≥64**	**≥200**
	**TL3086**		**128**	16	16	≥256	**16**	**8**	≥256	**128**	**≥64**	**≥200**
		**Breakpoints (S-R)**	4-16	4-16	2-16	1-4	0.25-1	0.5-2	4-16	4-16	2-4	

E. coli	DC90		≥256	32	64	≥256	64	32	≥256	128	≥64	≥200
	DC3411		64	32	16	1	64	32	4	2	4	100
	DC3539		64	128	≥256	0.5	64	16	128	128	16	100
	DC3599		≥256	128	≥256	0.5	≥256	≥256	≥256	≥256	≥64	100
	DC3737		≥256	≥256	≥256	128	≥256	≥256	≥256	≥256	≥64	100
	DC3806	MIC (μg/mL)	64	64	16	1	4	8	16	16	4	≥200
	DC3846		128	64	≥256	0.5	≥256	128	≥256	64	8	≥200
	DC4887		1	4	32	1	4	16	16	8	≥64	≥200
	DC5262		≥256	≥256	≥256	4	2	16	≥256	≥256	≥64	≥200
	DC5286		≥256	128	≥256	0.25	128	64	4	4	8	100
	DC7333		≥256	≥256	≥256	16	≥256	128	128	≥256	4	≥200
K. pneumoniae	FK20		≥256	128	≥256	16	≥256	64	8	≥256	4	100
	FK26		128	≥256	≥256	4	≥256	128	≥256	≥256	≥64	≥200
	FK150		0.0125	0.5	4	0.5	8	128	128	≥256	8	100
	FK169		1	16	0.5	4	2	1	1	64	≥64	50
	FK171		≥256	≥256	≥256	≥256	≥256	≥256	≥256	≥256	4	100
	FK591		≥256	≥256	64	1	≥256	≥256	≥256	64	≥64	100

**FK610**		≥256	≥256	≥256	**32**	**128**	**32**	**128**	**64**	**32**	**≥200**
**FK1342**		**128**	≥256	≥256	0.25	**1**	0.5	1	4	**≥64**	**≥200**
FK1986	**MIC (μg/mL)**	0.0125	0.25	0.0125	0.25	0.0125	0.025	2	1	**16**	**≥200**
**FK2066**		**≥256**	**≥256**	**128**	1	**≥256**	**≥256**	**≥256**	**≥256**	**≥64**	**100**
**FK2911**		**≥256**	**≥256**	**≥256**	**≥256**	**≥256**	**≥256**	**≥256**	**≥256**	**≥64**	**100**
**FK3789**		**≥256**	**≥256**	**≥256**	**128**	**≥256**	**≥256**	**≥256**	**≥256**	**≥64**	**100**
**FK3810**		0.0125	**128**	**≥256**	**32**	**≥256**	**128**	**≥256**	**≥256**	**≥64**	**100**
**FK3994**		**≥256**	**128**	**≥256**	**32**	**≥256**	**64**	**≥256**	**≥256**	**≥64**	**≥200**
**FK6556**		**64**	**64**	**64**	**16**	**4**	**8**	**16**	**16**	**32**	**≥200**
**FK6663**		**≥256**	**≥256**	**≥256**	**32**	**≥256**	**≥256**	**≥256**	**≥256**	**32**	**100**
**FK6696**		**≥256**	**64**	**≥256**	**128**	**≥256**	**64**	**≥256**	**≥256**	**≥64**	**100**

[a] Bolded strain number indicates multidrug resistant (MDR) strain.

[b] Bolded values point means resistance.

[c] S-R represents the susceptible (S) breakpoint to resistant (R) breakpoint, according to CLSI supplement M100 (30th edition) and EUCAST.

Abbreviations: GNB, Gram-negative bacteria; ATM, Aztreonam; CAZ, Ceftazidime; FEP, Cefepime; IMP, Imipenem; CIP, Ciprofloxacin; LVX, Levofloxacin; GEN, Gentamicin; TOB, Tobramycin, COL, colistin.

Table S2 Resistance mechanism of colistin in colistin-resistant GNB.

Species	Strains[a]	Resistance mechanism		
P. aeruginosa	TL1671	PmrB (V15I, P216S)		
	TL1722	PmrB (V199I, S257N)	ParR (R146H)	CprS (V181I, R209L)
	TL1736	PmrB (V185A)		
	TL1744	PmrB (V15I, G68S)		
	TL2204	PmrB (Y345H)		
	TL2294	PmrB (G179D, I349V)		
	TL2314		PhoQ (V260G)	
	TL2917	PmrB (G179D)	PhoQ (V260G)	
	TL2967	PmrB (D45E)		
	TL3008	PmrB (A190G)		
	TL3086	PmrB (S27R)		
E. coli	DC90	*mcr-1*		
	DC3411	*mcr-1*		
	DC3539	*mcr-1*		
	DC3599	*mcr-1*		
	DC3737	*mcr-1*		
	DC3806	*mcr-1*		
	DC3846	*mcr-1*		
	DC4887	*mcr-1*		
	DC5262	*mcr-1*		
	DC5286	*mcr-1*		
	DC7333	*mcr-1*		
K. pneumoniae	FK20	MgrB (K2E, F28C)		
	FK26	MgrB (M1V, K2E, F28C)		
	FK150	MgrB (K2E, F28C)	PhoQ (D150G)	
	FK169	MgrB (K2E, F28C)		
	FK171	MgrB (K2E, F28C)		

	FK591	MgrB (K2E, F28C)	PhoQ (D150G)	
	FK610	MgrB (K2E, F28C)		
	FK1342	MgrB (K2E, F28C)	PhoQ (D150G)	*mcr-1*
	FK1986	MgrB (K2E, F28C)	PhoQ (D150G)	
	FK2066		PhoQ (D150G)	
	FK2911		PhoQ (D150G)	
	FK3789	PmrB (R256G)		
	FK3810	PmrB (R256G)		
	FK3994	PmrB (R256G)	*mcr-1*	
	FK6556	PmrB (R256G)	*mcr-1*	
	FK6663	PmrB (R256G)	*mcr-1*	
	FK6696	PmrB (R256G)	PhoQ (L30Q)	

Table S3 Primers used for the detection of colistin resistance mechanisms.

Genes	Primer sequence (5′→3′)	Product size (bp)	Referenc[illegible]
mcr-1	F: GCTCGGTCAGTCCGTTTG	1626	(1)
	R: GAATGCGGTGCGGTCTTT		
pmrA	F: CGCAGGATAATCTGTTCTCCA	808	(2)
	R: GGTCCAGGTTTCAGTTGCAA		
pmrB	F: GCGAAAAGATTGGCAAATCG	659	(2)
	R: GGAAATGCTGGTGGTCATCTGA		
mgrB	F: AAGGCGTTCATTCTACCACC	253	(2)
	R: TTAAGAAGGCCGTGCTATCC		
phoP	F: GAGCGTCAGACTACTATCGA	912	(2)
	R: GTTTTCCCATCTCGCCAGCA		
phoQ	F: CCACAGGACGTCATCACCA	636	(2)
	R: AGCTCCACACCATATAGCTG		
pmrAB	F: TCACTGAAACGAGGCTGCCATGAG	2138	(3)
	R: CCAATGCGCAGGCTATCAGATATGT		
phoP	F: CGCCATATGAAACTGCTGGTAGTGGAA	690	(3)
	R: CGCCTCGAGCCGGCAGCGCTCGGTGAA		
phoQ	F: CGGCATATGATCCGTTCCCTGCGCATC	1359	(3)
	R: CGGCTCGAGGACTGTAGCGAAACGTATG		
parR	F: GCATATAATGCCAGCCGATT	1055	(4)
	R: ACCACCAGCAGGTTCTTGTC		
parR	F: CTATTCGCTGGTGGAAAAGC	1162	(4)

	R: GTTAAGCCTCCGCTGTCAAC		
cprR	F: CGCAGTATCCGAAGGAAGAA	1154	(4)
	R: CCCTTCCTCTTCCATCATCA		
cprS	F: TCTGATCCATACCCTGCACA	1002	(4)
	R: CTGTTCCTCGAGCAGTTCCT		

Reference

1. Liao W, Lin J, Jia H, Zhou C, Zhang Y, Lin Y, Ye J, Cao J, Zhou T. 2020. Resistance and Heteroresistance to Colistin in *Escherichia coli* Isolates from Wenzhou, China. Infect Drug Resist 13:3551-3561.
2. Ayerbe-Algaba R, Gil-Marques ML, Jimenez-Mejias ME, Sanchez-Encinales V, Parra-Millan R, Pachon-Ibanez ME, Pachon J, Smani Y. 2018. Synergistic Activity of Niclosamide in Combination with Colistin Against Colistin-Susceptible and Colistin-Resistant and *Klebsiella pneumoniae*. Front Cell Infect Microbiol 8:348.
3. Lin J, Xu C, Fang R, Cao J, Zhang X, Zhao Y, Dong G, Sun Y, Zhou T. 2019. Resistance and Heteroresistance to Colistin in *Pseudomonas aeruginosa* Isolates from Wenzhou, China. Antimicrob Agents Chemother 63.
4. Lister PD, Wolter DJ, Hanson ND. 2009. Antibacterial-resistant *Pseudomonas aeruginosa*: clinical impact and complex regulation of chromosomally encoded resistance mechanisms. Clin Microbiol Rev 22:582-610.
